# Physical Health Checks and Follow‐Up Care in Deprived and Ethnically Diverse People With Severe Mental Illness: Co‐Designed Recommendations for Better Care

**DOI:** 10.1111/hex.70005

**Published:** 2024-08-28

**Authors:** Easter Joury, Edward Beveridge, Judith Littlejohns, Angela Burns, Gemma Copsey, Justin Philips, Shanaz Begum, David Shiers, Carolyn Chew‐Graham, Charlotte Klass, Jackie Chin

**Affiliations:** ^1^ Institute of Dentistry Queen Mary University of London London UK; ^2^ Royal London Dental Hospital Barts Health NHS Trust London United Kingdom; ^3^ UCLPartners London UK; ^4^ Tower Hamlets North East London Integrated Care Board London UK; ^5^ Healthy Young Adults, London Borough of Tower Hamlets London UK; ^6^ NHS North East London, North East London Health and Care Partnership London UK; ^7^ Tower Hamlets Early Detection Service London UK; ^8^ Greater Manchester Mental Health NHS Foundation Trust University of Manchester Manchester UK; ^9^ Division of Psychology and Mental Health University of Manchester Manchester UK; ^10^ Primary Care and Health Sciences, School of Medicine University of Keele Newcastle UK; ^11^ School of Medicine, Faculty of Medicine and Health Sciences Keele University Newcastle UK; ^12^ NHS England London Region London UK; ^13^ Department of Health and Social Care Office for Health Improvement and Disparities, London Region London UK

**Keywords:** co‐design, health inequities, health promotion, integrated care pathways, oral health, physical health, primary care, severe mental illness

## Abstract

**Background:**

There is wide variation in premature mortality rates in adults with severe mental illness (SMI) across London, with Tower Hamlets (a highly deprived and ethnically diverse area) scoring the highest.

**Objective:**

To identify examples of best practice and co‐design recommendations for improving physical health checks and follow‐up care amongst people with SMI in Tower Hamlets.

**Methods:**

Data were collected through online questionnaires (using SMI physical health best practice checklists), one‐on‐one interviews (*n* = 7) and focus groups (*n* = 3) with general practices, secondary mental health services, commissioners and leads of community services and public health programmes, experts by experience and community, voluntary and social enterprise organisations in Tower Hamlets. Data were analysed using deductive and inductive thematic analysis.

**Results:**

Twenty‐two participants representing 15 general practices (out of 32), secondary mental health services, commissioners and public health leads completed the online questionnaires. Twenty‐one participants took part in interviews and focus groups. Examples of best practice included cleaning and validating the SMI register regularly by general practices, knowing the number of patients who had been offered and/or received physical health checks, having clear pathways to community and specialist care services, using various communication methods and having a key performance indicator (KPI) for tailored smoking cessation services for people with SMI. Recommendations included adopting evidence‐informed frameworks for risk stratification and utilising the wider primary care workforce with specific training to follow up on results, offer interventions and support navigating pathways and taking up follow‐up care. Incentivising schemes were needed to deliver additional physical health check components such as oral health, cancer screening, Covid‐19 vaccination and sexual health checks. Including KPIs in other community services' specifications with reference to SMI people was warranted. Further engagement with experts by experience and staff training were needed.

**Conclusion:**

The present initiative identified best practice examples and co‐designed recommendations for improving physical health checks and follow‐up care in deprived and ethnically diverse people with SMI.

**Patient or Public Contribution:**

This initiative was supported by three experts with experience, and two community organisations, who were involved in data curation and interpretation, development of recommendations and/or dissemination activities including writing this manuscript.

## 1 Introduction

People with severe mental illness (SMI) experience some of the worst social and physical health inequalities [1]. They die 15 to 20 years earlier than the general population. Two‐thirds of this premature mortality is due to preventable and treatable chronic physical health conditions such as cardiovascular, respiratory and liver diseases, diabetes and some cancers [2].

Over the years, the difference in premature mortality between people with SMI and the general population has been increasing in England. Analyses conducted by the Office for Health Improvement and Disparities (OHID) based on data from 2016 to 2018 showed that people with SMI were 4.5 times more likely to die prematurely than their counterparts with no SMI in England [2]. This excess premature mortality gap is significantly wider than that reported for the period 2015–2017 [2]. Physical multimorbidity tends to build quickly in a potentially young SMI population [3].

The primary care team plays a critical role in addressing the health inequalities experienced by people with SMI and their carers [4]. At the centre of this role lies improving the coverage, timeliness and quality of physical health care for people with SMI through physical health checks and evidence‐informed follow‐up interventions. The physical health checks include six core components, dictated by the Quality and Outcomes Framework (QOF), as follows: blood pressure, body mass index, lipid profile, blood glucose or HbA1c test, alcohol consumption and smoking status [5]. QOF is an incentive programme for resourcing and rewarding good practice in all general practices in England [5]. The updated Lester resource emphasises the importance of intervening following the physical health checks, including evidence‐based cardiovascular risk assessment and management, support for tobacco cessation and advice about diet, alcohol and physical activity [6, 7, 8, 9, 10, 11]. However, the uptake and adherence to guidelines on screening, monitoring and prompt interventions on modifiable risk factors amongst people with SMI remains a major area for improvement [12].

There have been policy solutions in England aimed at improving physical health amongst people with SMI since at least 2012, when the Royal College of Psychiatrists' National Audit of Schizophrenia (NAS, now National Clinical Audit of Psychosis or NCAP) found that only 29% of patients with SMI had received a full physical health check [13, 14]. This led to an NHS England Commissioning for Quality and Innovation (CQUIN) incentive scheme from 2016 to 2019 to encourage specialist care providers to provide physical health checks [15]. There is evidence that the number of physical health checks improved during the period [16].

The NHS Long Term Plan and Community Mental Health Framework have continued and expanded the ambition of the CQUIN [17, 18]. The former outlined a commitment to 390,000 health checks a year from 2023 to 2024 for people with SMI, including follow‐up interventions. In primary care, general practices are incentivised via the QOF to carry out physical health checks for people with SMI [5].

The Covid‐19 Mental Health and Wellbeing Recovery Action Plan made holistic and joined‐up support for people with SMI a priority area for action [19]. NHS England and NHS Improvement have invested £14 million to work across primary and secondary care services with voluntary and community sector partners [19]. The plan aimed to deliver tailored outreach and engagement for people with SMI to increase their uptake of physical health checks. Additionally, the national Core20Plus5 initiative to reduce health inequalities made increasing the uptake of SMI physical health checks one of its five priority clinical areas [20].

Besides the above policy solutions, interventions to improve the practice of physical health checks and follow‐up care were developed [21]. This included developing frameworks for risk stratifications and intensive follow‐up. One example of such interventions is UCLP Primrose Framework, which has three components: (i) risk stratification and search tools to identify patients at highest clinical risk or not engaged with care; (ii) support to improve the annual SMI physical health checks including clinical review to best treat the most important risk factors (e.g., blood pressure and lipids); and (iii) materials and training for offering structured behaviour change interventions and peer support to ensure both physical and mental health improvements [10, 21].

In London, UK, there is wide variation in premature mortality in adults with SMI across boroughs, with Tower Hamlets having the highest rate [2]. A directly standardised rate (DSR) of 146.9 per 100,000 was reported in this borough. This showed a significantly higher rate compared to England's average and a twofold increase in mortality rate compared to other boroughs in London such as Richmond upon Thames [2]. General practitioner (GP) registers showed that the adult population in Tower Hamlets had higher levels of SMI (1.34%, 4334 patients) than London or England [22]. The percentage of physical health checks amongst people with SMI in Tower Hamlets was 43.4 by the beginning of 2022/23 [23]. These stark inequalities could be explained by the complex intersectionality between SMI, deprivation and ethnicity. Tower Hamlets is characterised by high levels of deprivation and a highly ethnically diverse population, with over half from minority ethnic backgrounds.

To respond to the above, the Local Authority in Tower Hamlets incorporated improving physical health in its Adult Mental Health Strategy. One of the action areas is increasing the number of people with SMI who access physical health checks and reaching out to those in most need through more targeted initiatives. Yet, there remains a need to drive improvement in the physical health checks and follow‐up care in Tower Hamlets through collaboration amongst key stakeholders: primary care, secondary mental health services, community services, public health programmes, *experts by experience* and voluntary, community and social enterprise (VCSE) organisations. Such collaboration is key to improving physical health and tackling the stark inequalities in premature mortality experienced by people with SMI living in deprived and ethnically diverse communities [24]. Thus, the aim of the present collaborative initiative was to identify examples of best practice and co‐design recommendations to improve the quality of physical health checks and follow‐up care amongst people with SMI in Tower Hamlets.

## 2 Materials and Methods

The current initiative was exempted from requiring ethics approval, as it was considered an engagement and quality improvement activity.

Stakeholder mapping was carried out and a working group comprising representatives from various stakeholders was formed to lead the initiative. Data were collected using online questionnaires, seven online and in‐person one‐on‐one interviews and three focus groups (with 4–6 participants each).

The online questionnaires were developed based on two checklists of best practice in physical health checks and follow‐up care to assess current practice, identify areas for improvement and create actions to better current practices [25]. The primary care and secondary mental health services questionnaire included 30 items of best practice under eight domains: governance and leadership, data, engagement with people registered with SMI, physical health checks, the offer of brief interventions, pathways into community services, pathways into acute/specialist services and staff training (Appendix S1). It included one item of best practice on oral health (whether oral health was included in the physical health checks offered to people with SMI). Referral to dental services was added to the best practice item on pathways to specialised services (Appendix S1). The Local Authority questionnaire included 27 items of best practice under seven domains: the commissioning landscape across the Local Authority, system governance and leadership, commissioning and finances, data analysis and flows, pathways, interventions and service user experience (Appendix S2). The presence of specific references to people with SMI in oral health promotion programmes and the availability of co‐produced oral health resources were added to the corresponding best practice items on commissioning landscape, pathways and co‐production of resources and information (Appendix S2). Participants were asked to score each item of best practice as fully, partially, limitedly or not implemented. A ‘do not know’ option was added to the answer options. Under each item, an open‐ended question was added to allow participants to comment on barriers and enablers to implementing that area of best practice, share their own examples of best practice and provide any additional comments.

The two questionnaires were then piloted, revised to enhance clarity and distributed to all Tower Hamlets general practices (*n* = 32), primary care networks, East London Foundation Trust (secondary mental health services in Tower Hamlets), community and mental health services commissioners and public health leads in the Local Authority. Each general practice was asked to select a representative to complete the questionnaire, who could be a GP, practice manager or physical health checks lead. Seven one‐on‐one interviews and three focus groups were carried out online or in person by the lead author with different stakeholders including people with lived experience and local CVSE organisations. This helped explore examples of best practices and challenging areas for improvement in more depth, and co‐design recommendations. The lead author also attended national, regional and local events, webinars, and networks and delivery groups meetings, and contacted other SMI physical health and public health leads to learn about examples of best practice in other areas and regions to inform the recommendations of the present initiative.

Data collection took place between September and October 2022. Quantitative data on best practice implementation was grouped into three categories: (i) fully, (ii) ‘partially, limited or no’ and (iii) ‘I do not know’ (Appendices S1 and S2). Qualitative data collected by the online questionnaires, interviews and focus groups were analysed through deductive and inductive thematic analysis. Themes were set a priori using the above‐mentioned domains of best practice in the two questionnaires. Furthermore, the new themes that emerged from the data were reported. Generating the recommendations was based on the items of best practice that the majority of respondents identified as partially, limitedly or not implemented. These were further developed and contextualised using qualitative data on barriers and enablers of implementation reported in the online questionnaire and collected through one‐on‐one interviews and focus groups.

The findings and recommendations were shared and discussed with stakeholders via an online event hosted by the Community Education Provider Network (CEPN), to seek further contributions, and facilitate the ownership of the co‐designed recommendations and change management. *Experts by experience* led an activity in this CEPN event, where they planned and delivered an oral presentation followed by an interactive questions and answers session on patient experience of physical health checks and follow‐up care. They highlighted examples of best practice and areas for improvement and proposed solutions.

## 3 Results

Nineteen respondents completed the primary care and secondary mental health services questionnaire. They included staff from 15 out of 32 general practices in Tower Hamlets (12 GPs, one lead nurse, one healthcare assistant and one pharmacist trainee), one primary care network manager and three secondary mental health services staff (one community psychiatric nurse and two occupational therapists). Three respondents completed the Local Authority online questionnaire, representing the integrated commissioning team, and the leads of community services and public health programmes.

Quantitative data are presented in Appendices S1 and S2. The following summarises qualitative data.

### 3.1 Examples of Best Practice and Co‐Designed Recommendations for Primary and Secondary Mental Health Services

**Figure 1:**
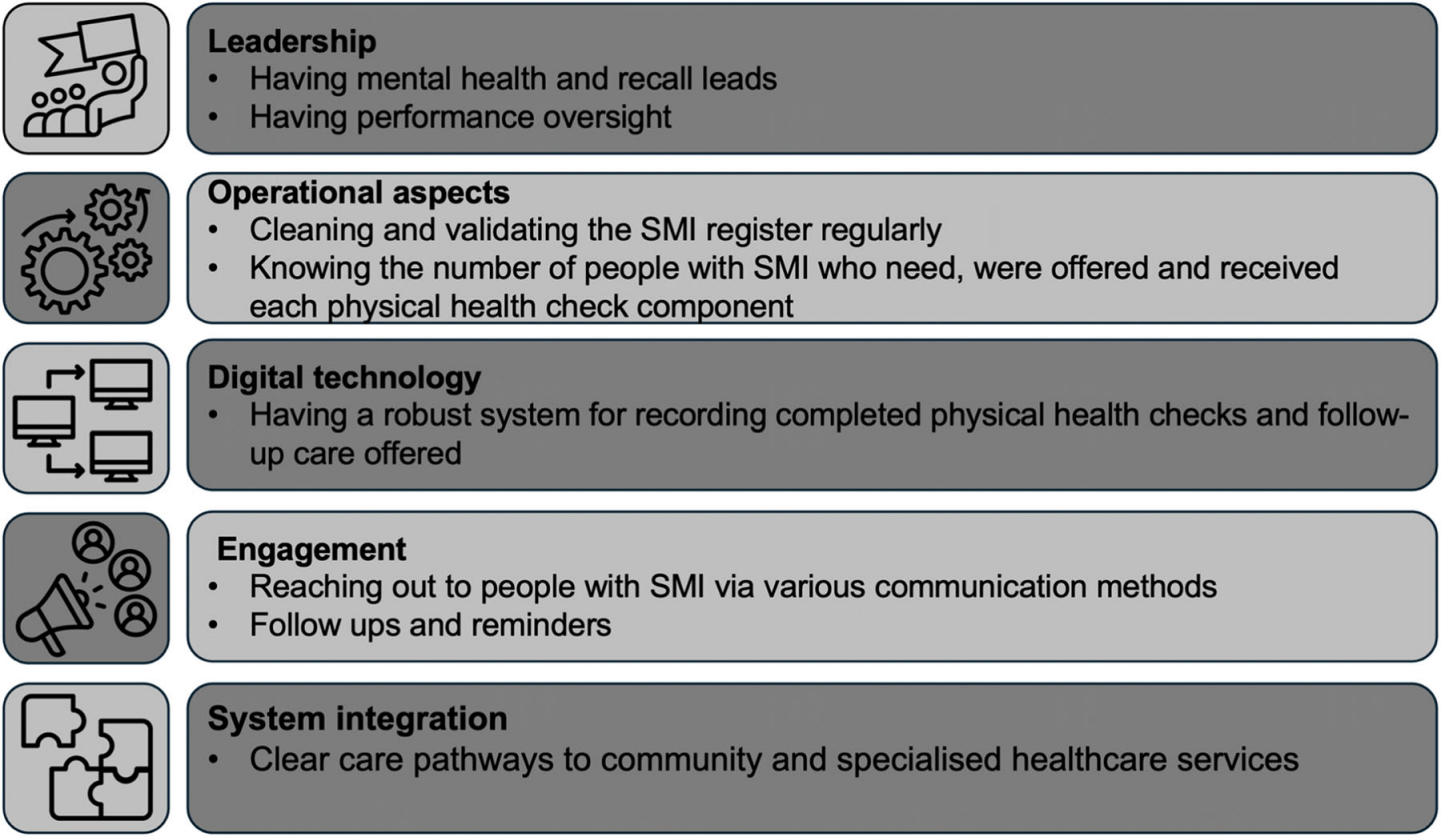
Examples of best practice by primary care and secondary mental health services on physical health checks and follow‐up care amongst people with severe mental illness. SMI, severe mental illness.

**Figure 2:**
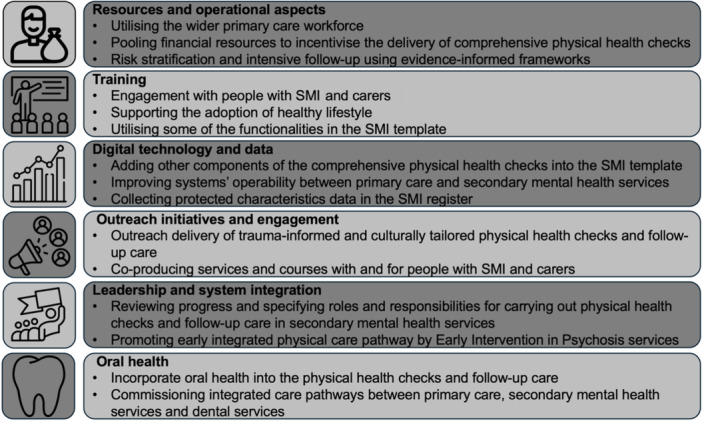
Co‐designed recommendations for primary care and secondary mental health services for physical health checks and follow‐up care amongst people with severe mental illness. SMI, severe mental illness.

A summary of best practice examples and co‐designed recommendations for primary care and secondary mental health services is presented in Figures 1 and 2.

#### 3.1.1 Examples of Best Practice

##### 3.1.1.1 Leadership

Most general practices had dedicated mental health and call/recall leads. The majority of general practices reported having a plan and performance oversight process in place to monitor the delivery of the upper threshold of all physical health checks in the SMI QOF incentives (at a component level).

##### 3.1.1.2 Operational Aspects

Most general practices cleansed and validated their SMI register regularly, keeping it up to date. Together with mental health services, they knew the number of patients needing physical health checks to meet their target, those who had been offered these checks and the percentage of patients who have had the core physical health check components (at a component level).

##### 3.1.1.3 Digital Technology

Most general practices and secondary mental health services had a robust system for recording completed physical health checks and follow‐up interventions offered.

##### 3.1.1.4 Engagement

A variety of communication methods were used to invite/remind patients on the SMI register regarding physical health checks (such as using letters and text messages, and phone calls with interpreters when needed). Some general practices had ‘Did Not Attend’ (DNA) process of sending three follow‐ups or reminders.

##### 3.1.1.5 System Integration

Most general practices and secondary mental health services had clear pathways for referring patients to community services such as social prescribing, smoking cessation services, alcohol and drug misuse services and some weight management services. They also had clear pathways for sign‐posting patients to cancer screening programmes and Covid‐19 vaccination programmes, referring them to specialist consultant‐led services, and follow‐up on their long‐term conditions in Long‐Term Conditions review clinics run by general practices.

#### 3.1.2 Areas for Improvement and Co‐Designed Recommendations

##### 3.1.2.1 Resources and Operational Aspects

General practices could improve physical health checks and follow‐up care by risk‐stratifying patients on the SMI register based on their clinical risks and level of engagement. They could also improve by utilising the members of the wider primary care team to follow up on results, offer interventions and support patients in navigating pathways and taking up needed acute, specialist and community services (including social prescribing). Social prescribing involves referral to non‐medical services to help people identify their social needs and develop ‘well‐being’ action plans to promote, establish or re‐establish integration and support in their communities, with the aim of improving personal well‐being [26].

Suggestions to facilitate the above included adopting evidence‐informed frameworks for risk stratifications and intensive follow‐up such as UCLP Primrose Framework [10, 21]. The need to utilise the members of the broader primary care team could be addressed by the utilisation of general healthcare professionals and workers with specific training, such as health and well‐being coaches, healthcare assistants, social prescribers and community connectors. These staff members are employed in primary care under the Additional Roles Reimbursement Scheme (ARRS) [27]. The ARRS provides funding to Primary Care Networks in England to recruit additional staff into specified roles, enhance the capacity of primary care services to meet the growing healthcare demand, deliver a broader range of integrated services and adopt a more preventative and community‐based care.

An incentivising scheme was suggested to deliver additional components of physical health checks and brief follow‐up interventions, such as oral health advice (providing oral hygiene support, mouth cancer early detection and facilitating access to dental care as needed), cancer screening (checking and supporting the uptake of cancer screening programmes), immunisation programmes (e.g., checking and supporting the uptake of Covid‐19 vaccination) and sexual health assessment and advice. Such an incentivising scheme could be developed and enacted by the Integrated Care Board (ICB), a statutory NHS organisation responsible for developing a plan to meet the health needs of the population, managing the NHS budget and arranging for the provision of health services in a geographical area [28]. The ICB could pool funds from mental health services, primary care, complex care and dentistry funding to create the above incentivising scheme.

##### 3.1.2.2 Training

Training focussed on Making Every Contact Count (MECC) was reported. MECC is an approach to behaviour change that uses the millions of day‐to‐day interactions that organisations and individuals have with other people to support them in making positive changes to their physical and mental health and well‐being. It supports the opportunistic delivery of consistent and concise healthy lifestyle information and enables individuals to engage in conversations about their health [29]. MECC training was requested to be available on demand and be part of protected learning time. Using NHS England Workforce Training and Education MECC e‐learning and resources was suggested to meet this training need. Further training was also requested on utilising some of the SMI template functionalities. Training needs were highlighted by healthcare assistants (and other physical health checks staff) regarding engagement with SMI patients and carers, and supporting the adoption of healthier lifestyles including oral hygiene.

##### 3.1.2.3 Digital Technology and Data

The need for adding additional components such as oral health and sexual health into the SMI template was highlighted. The use of the 2023 Lester resource, to address the cardiometabolic changes commencing within weeks of antipsychotic drug initiation, was suggested [6]. The need to collect full data on protected characteristics in the SMI register was also noted. Furthermore, the need for solutions to overcome the challenges of systems' interoperability and use of different electronic patient records was voiced by staff in secondary mental health services. Digital solutions such as the use of similar SMI templates and the same coding system were suggested.

##### 3.1.2.4 Outreach Initiatives and Engagement

The need for outreach service delivery models was emphasised. For example, one of the recommendations included developing outreach weight management initiatives, which could include offering drop‐in assessments and physical activity sessions at a community centre. Such outreach initiatives should be trauma informed and culturally tailored to increase uptake in local marginalised communities (particularly women from minority ethnic backgrounds). New service models were also suggested. This included a voucher scheme, where vouchers are distributed to people with SMI to encourage taking up physical health checks and follow‐up care.

There was a need for more local engagement with people with SMI and their carers to (i) understand better barriers and facilitators to taking up physical health checks and follow‐up care, (ii) co‐create culturally and logistically appropriate services tailored to their needs and (iii) provide input into the business case for incentivising physical health checks. Co‐designing online educational resources for people with SMI and carers was proposed.

##### 3.1.2.5 Leadership and System Integration

There was a need to review progress on the physical health checks work with secondary mental health services. This should include clarifying whose responsibility it is to carry out the physical health checks within secondary mental health services. Moreover, healthcare assistants were operating across primary care and secondary mental health services. They invariably found such work across multiple teams and organisational boundaries challenging. Furthermore, promoting an early physical care pathway led by Early Intervention in Psychosis services, working collaboratively across the system to avoid service disconnects in the first few years, was recommended. Early Intervention in Psychosis services are multidisciplinary community mental health services that provide treatment and support to people experiencing a first episode of psychosis or at high risk of developing psychosis [30]. This support typically continues for 3 years and includes a full range of evidence‐based treatments including pharmacological, psychological, social, occupational and educational interventions.

##### 3.1.2.6 Oral Health

The need for incorporating oral health into the physical health checks and follow‐up care was highlighted. Commissioning integrated care pathways between primary care and secondary mental health services and dental services was warranted.

### 3.2 Examples of Best Practice and Co‐Designed Recommendations for Community Services and Public Health Programmes

**Figure 3:**
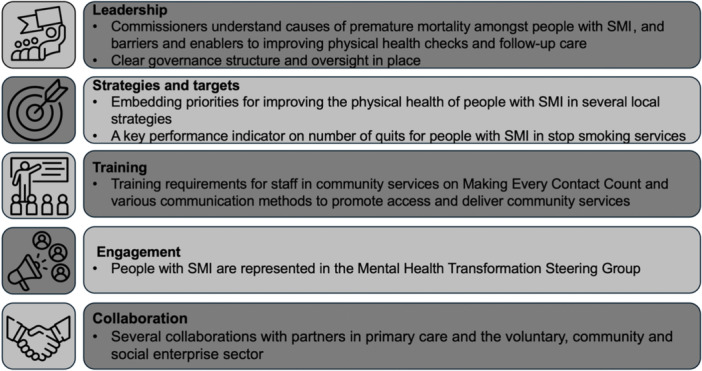
Examples of best practice by community services and public health programmes to improve the physical health amongst people with severe mental illness. SMI, severe mental illness.

**Figure 4:**
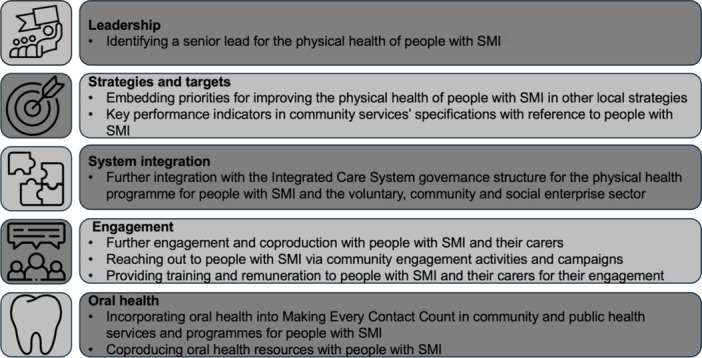
Co‐designed recommendations for community services and public health programmes to improve the physical health amongst people with severe mental illness. SMI, severe mental illness.

A summary of best practice examples and co‐designed recommendations for community services and public health programmes commissioned by the Local Authority is presented in Figures 3 and 4. Themes on leadership, strategies and targets, system integration, collaboration, engagement, training and oral health were identified.

### 3.3 Initial Impacts of This Collaborative Initiative

#### 3.3.1 Individual Impacts

Excellent feedback from *experts by experience* was received. It highlighted the impact of the present initiative on them in terms of feeling empowered, heard and able to make a difference to the physical health and well‐being of people with SMI. One example of the feedback was: ‘It was a pleasure to meet everyone and to be part of such a great event. Thank you for the opportunity … to be part of it alongside such great speakers—I found all the presentations very informative and useful. I'll look forward to our paths crossing again soon!’. The feedback also emphasised the need for establishing, funding and building the capacity of local SMI *experts by experience*.

#### 3.3.2 Project Impacts

The Tower Hamlets Mental Health Partnership Board started translating the co‐designed recommendations from this collaborative initiative into various workstreams to drive changes in policy and practice. This included securing new funding and recruiting primary care healthcare assistants and secondary mental health services peer support workers. A co‐produced ‘UCLP Primrose’ Primary Care Network pilot was set up to support people with SMI to build behavioural strategies to improve their cardiovascular health. Furthermore, a ‘Rethink’ physical activity programme was launched. Further new funding was secured to set up a community‐based project to increase the uptake of follow‐up interventions in SMI people from minority ethnic backgrounds.

## 4 Discussion

The initiative described in this paper identified examples of best practices and co‐designed recommendations for improving physical health checks and follow‐up care in deprived and ethnically diverse people with SMI. Furthermore, the findings of the present initiative were used to inform the recent national guidance on improving the physical health of people living with SMI [31].

Our identified areas for improvement and co‐designed recommendations are supported by previous findings reported in the literature. For example, the need for system integration and incorporating oral health into the physical health care of people with SMI was repeatedly reported in the literature [32, 33]. Previous studies highlighted the impact of financial incentives on the uptake of physical health checks amongst people with SMI [34, 35]. For example, Matias et al., using a difference‐in‐difference analysis at a patient level, assessed the impact of removing and reintroducing QOF incentives on the uptake of three physical health check components (body mass index, lipid profile and alcohol consumption). They found an immediate drop in the uptake of these components following the removal of QOF incentives, which was immediately reversed when the incentives were reinstated. The literature also reported positive impacts of training and utilising the wider primary care and mental health workforce on the uptake and quality of physical health checks for people with SMI [33, 36]. For example, training primary care nurses and community psychiatric nurses in secondary care to carry out physical health checks for people with SMI has led to an increase in the uptake and quality of physical health checks and lifestyle advice given to this group of people. It also ensured working systematically and comprehensively [33]. A hybrid delivery model of physical health checks, which included outreach and domiciliary care, was identified as a key element that increased the uptake of physical health checks amongst the users of Early Intervention in Psychosis services [35]. Simplifying the system used to record physical health checks and integrating it with the wider assessment carried out upon admission to psychiatric inpatient wards showed a significant increase in the uptake of physical health checks [37].

This initiative has several strengths. One of its strengths is related to the various data collection methods used. For example, the use of online questionnaires allowed reaching out to all general practices in a shorter timeframe. Findings from the online questionnaires were triangulated and contextualised using interviews and focus groups with different stakeholders, including *experts by experience* and local VCSE organisations. This allowed exploring some findings in more depth and co‐designing recommendations. A further strength is related to the change management strategy adopted, where a CEPN event was organised to discuss findings and recommendations, ensuring that all voices (particularly the voices of *experts by experience*) were heard and represented appropriately. It brought together all key stakeholders who can input and take forward the co‐designed recommendations. This allowed mediating and forging new collaborations to bring about improvement in the physical health checks and follow‐up care in a deprived and ethnically diverse SMI population.

The initiative is not without limitations. One of the limitations was related to the length of the online questionnaires. These could be shortened by merging some questions that are covering similar topics. For example, questions around engagement with people with SMI could be merged.

A key policy implication of the current initiative's findings is the need to develop a national financial model to incentivise comprehensive physical health checks beyond the six core components. In terms of practice implications, there is a need to co‐produce all services related to delivering physical health checks and follow‐up care to deprived and ethnically diverse people with SMI. This ensures that these services are both culturally and logistically appropriate as well as tailored to their needs and challenging living conditions.

## 5 Conclusion

The current collaborative initiative identified best practice examples and co‐designed recommendations for improving the physical health checks and follow‐up care in deprived and ethnically diverse people with SMI.

## Author Contributions

**Easter Joury:** conceptualisation, methodology, software, project administration, investigation, data curation, validation, formal analysis, visualisation, writing–original draft, writing–review and editing. **Edward Beveridge:** methodology, validation, writing–original draft, writing–review and editing. **Judith Littlejohns:** methodology, data curation, validation, writing–review and editing. **Angela Burns:** methodology, data curation, validation, writing–review and editing. **Gemma Copsey:** methodology, validation, writing–review and editing. **Justin Philips:** data curation, validation, writing–review and editing. **Shanaz Begum:** data curation, validation, writing–review and editing. **David Shiers:** writing–original draft, writing–review and editing. **Carolyn Chew‐Graham:** writing–review and editing. **Charlotte Klass:** writing–review and editing. **Jackie Chin:** conceptualisation, methodology, data curation, formal analysis, writing–original draft, writing–review and editing.

## Conflicts of Interest

David Shiers is an expert advisor to the NICE Centre for Guidelines; the views expressed in this paper are those of the authors and not those of NICE. Carolyn Chew‐Graham is the Editor‐in‐Chief, *Health Expectations*, and is partly funded by WM ARC.

## Supporting information

Supporting information.

Supporting information.

## Data Availability

The data that support the findings of this study are available from the corresponding author upon reasonable request.
