# Supplementary material for: Physical Health Checks and Follow‐Up Care in Deprived and Ethnically Diverse People With Severe Mental Illness: Co‐Designed Recommendations for Better Care
Source: Health Expect. 2024 Aug 28;27(5):e70005. doi: 10.1111/hex.70005 (PMC11350427; doi:10.1111/hex.70005)
Supplement: Supplementary file 1 — Supporting information. [file HEX-27-e70005-s001.docx]

**Appendix 1: Primary care and secondary mental health services questionnaire and response counts**

You can respond anonymously to this survey – you do not have to give your name or contact details.

By completing this survey you are agreeing to your opinions being included in the decision making process about future recommendations.

| About you |
| --- |
| Your name (optional) |
| You work at (tick all that apply)   - GP practice - PCN - East London Foundation Trust |
| GP practice / PCN / Trust name (optional) |
| Your role |

| **Domains of best practice** | **Best practice implementation** | | |
| --- | --- | --- | --- |
|  | Fully (n) | Partially / Limited / No (n) | I do not know (n) |
| **Governance and leadership** | | | |
| 1.a. There is a dedicated mental health lead within the GP practice (for primary care only) | 15 | 4 | 0 |
| 1.b. There is a dedicated health check lead within the GP practice / PCN / Trust | 8 | 8 | 3 |
| Please add any additional comments (including related barriers, enablers and examples of best practice) | | | |
| **Data – Better understanding the people on your severe mental illness register** | | | |
| 2. The practice / PCN / Trust maintains a severe mental illness register that is regularly cleansed/validated and includes full data on protected characteristics | 15 | 4 | 0 |
| Please add any additional comments (including related barriers, enablers and examples of best practice) | | | |
| 3. Patients on the severe mental illness register have been risk stratified, identifying those in need of most intensive support based on the clinical risks to their physical health (using physical health diagnoses, QRISK3 scores and/or UCLP-Primrose priority group as indicators) and their level of engagement in monitoring/treatment | 7 | 11 | 1 |
| Please add any additional comments (including related barriers, enablers and examples of best practice) | | | |
| 4. The practice / PCN / Trust knows the number of patients on the severe mental illness register needing physical health checks in order to meet current target | 18 | 1 | 0 |
| Please add any additional comments (including related barriers, enablers and examples of best practice) | | | |
| 5. The practice / PCN / Trust has a plan and performance oversight process in place to monitor delivery of the upper threshold (90%) of all of the physical health checks in severe mental illness Quality and Outcome Framework incentives | 12 | 4 | 3 |
| Please add any additional comments (including related barriers, enablers and examples of best practice) | | | |
| 6. The practice / PCN / Trust knows the % of patients on the severe mental illness register who have had offered a health check | 11 | 4 | 4 |
| Please add any additional comments (including related barriers, enablers and examples of best practice) | | | |
| 7. The practice / PCN / Trust knows which population groups on the severe mental illness register are least likely to attend for their physical health checks | 4 | 13 | 2 |
| Please add any additional comments (including related barriers, enablers and examples of best practice) | | | |
| 8. The practice / PCN / Trust knows the % of patients on the severe mental illness register who have had a full health check in the last 12 months | 16 | 3 | 0 |
| Please add any additional comments (including related barriers, enablers and examples of best practice) | | | |
| 9. The practice / PCN / Trust knows the % of patients on the severe mental illness register that have had a partial health check in the last 12 months | 10 | 8 | 1 |
| What does the practice / PCN / Trust do about patients on the severe mental illness register that have had a partial health check in the last 12 months? | | | |
| Please add any additional comments (including related barriers, enablers and examples of best practice) | | | |
| 10. The practice / PCN / Trust knows the % of patients on the severe mental illness register that have had each of the individual checks in the last 12 months: | | | |
| a) a measurement of weight (BMI or BMI + waist circumference) | 13 | 5 | 1 |
| b) a blood pressure and pulse check (diastolic and systolic blood pressure recording or diastolic and systolic blood pressure + pulse rate) | 14 | 4 | 1 |
| c) a blood lipid including cholesterol test (cholesterol measurement or QRISK® measurement) | 14 | 4 | 1 |
| d) a blood glucose test (blood glucose or HbA1c measurement) | 13 | 5 | 1 |
| e) an assessment of alcohol consumption | 13 | 5 | 1 |
| f) an assessment of smoking status | 12 | 6 | 1 |
| g) an assessment of nutritional status, diet and level of physical activity (nutrition/diet status + physical activity/exercise) status | 12 | 6 | 1 |
| h) an assessment of use of illicit substance/nonprescribed drugs (substance misuse status) | 11 | 7 | 1 |
| i) medicines reconciliation | 11 | 6 | 2 |
| Please add any additional comments (including related barriers, enablers and examples of best practice) | | | |
| 11. The practice / PCN / Trust includes oral / dental health in the physical health checks offered to people with severe mental illness | 2 | 9 | 8 |
| Please add any additional comments (including related barriers, enablers and examples of best practice) | | | |
| **Engagement with patients registered with severe mental illness** | | | |
| 12. The practice / PCN / Trust uses data to engage with and target those population groups on the severe mental illness register who are least likely to take up the offer of a physical health check | 7 | 9 | 3 |
| Please add any additional comments (including related barriers, enablers and examples of best practice) | | | |
| 13. The practice / PCN / Trust knows the barriers and facilitators to enable patients to take up their physical health checks | 4 | 14 | 1 |
| Please add any additional comments (including related barriers, enablers and examples of best practice) | | | |
| 14. The practice / PCN / Trust has a plan for prioritising equity of access by protected characteristics | 7 | 7 | 5 |
| Please add any additional comments (including related barriers, enablers and examples of best practice) | | | |
| 15. The practice / PCN / Trust has co-produced culturally appropriate services to suit the needs of patients (including reasonable adjustments and domiciliary / outreach provision of physical health checks) | 5 | 11 | 3 |
| Please add any additional comments (including related barriers, enablers and examples of best practice) | | | |
| 16. The practice / PCN / Trust involves and provides support for carers / family members / friends to enable patients to take up their physical health checks | 7 | 9 | 3 |
| Please add any additional comments (including related barriers, enablers and examples of best practice) | | | |
| **Physical health checks** | | | |
| 17. A system is in place to ensure that everyone who is eligible for a physical health check is offered one | 15 | 4 | 0 |
| What system is in place (if any) to ensure that everyone who is eligible for a physical health check is offered one? | | | |
| Please add any additional comments (including related barriers, enablers and examples of best practice) | | | |
| 18. A variety of communication methods are used in order to reach your patient cohort who are eligible for a physical health check, including non-English speakers | 13 | 5 | 1 |
| Please add any additional comments (including related barriers, enablers and examples of best practice) | | | |
| 19. A system is in place that follows up those who do not take up the offer of the physical health checks | 11 | 6 | 2 |
| Please add any additional comments (including related barriers, enablers and examples of best practice) | | | |
| 20. Information for those on the severe mental illness register on physical health checks is available in a range of formats | 14 | 3 | 2 |
| Please add any additional comments (including related barriers, enablers and examples of best practice) | | | |
| 21. Text messaging or other innovative methods are used to invite/remind people of their physical health checks | 13 | 5 | 1 |
| Please add any additional comments (including related barriers, enablers and examples of best practice) | | | |
| 22. The practice / PCN / Trust has a robust system for recording completed physical health checks using a structured template e.g. the Bradford template | 14 | 2 | 3 |
| Please add any additional comments (including related barriers, enablers and examples of best practice) | | | |
| 23. The AUDIT-C tool for alcohol is embedded into the local electronic patient record system to drive screening and reporting | 15 | 2 | 2 |
| Please add any additional comments (including related barriers, enablers and examples of best practice) | | | |
| 24. The ASSIST-Lite screening tool for substance misuse is embedded into the local electronic patient record system to drive screening and reporting | 4 | 6 | 9 |
| Please add any additional comments (including related barriers, enablers and examples of best practice) | | | |
| **Offer of brief interventions** | | | |
| 25. The Making Every Contact Count framework is used to offer brief interventions relating to:  • alcohol  • drug misuse  • diet  • physical activity  • stop smoking  • lowering blood pressure | 10 | 4 | 5 |
| Please add any additional comments (including related barriers, enablers and examples of best practice) | | | |
| 26. Offers of interventions and interventions delivered are recorded in patient records | 15 | 4 | 0 |
| Please add any additional comments (including related barriers, enablers and examples of best practice) | | | |
| **Pathways into community services for those identified as requiring support via their physical health check** | | | |
| 27. Following a physical health check, there is a clear pathway for patients of the severe mental illness register into relevant community services: | | | |
| Social prescribing provision | 14 | 4 | 1 |
| Stop smoking services | 15 | 4 | 0 |
| Alcohol services | 17 | 2 | 0 |
| Drug misuse services | 17 | 2 | 0 |
| Weight management services | 10 | 7 | 2 |
| Physical activity services including exercise on referral schemes | 12 | 7 | 0 |
| How do the practice / PCN / Trust staff support patients with severe mental illness to access relevant community services? | | | |
| Please add any additional comments (including related barriers, enablers and examples of best practice) | | | |
| **Pathways into acute/specialist services for those identified as requiring support via their physical health checks** | | | |
| 28. Following a physical health check, there is a clear pathway for patients of the severe mental illness register into relevant specialist services and patients are supported to access: | | | |
| Cancer screening programmes (breast, bowel, cervical) | 14 | 4 | 1 |
| Specialist consultant led services, e.g. cardiology, endocrinology | 14 | 4 | 1 |
| LTC clinics e.g. diabetes and asthma annual reviews | 16 | 3 | 0 |
| Dental services | 4 | 11 | 4 |
| Other (please specify) | 5 | 3 | 11 |
| How do the practice / PCN / Trust staff support patients with severe mental illness to access relevant specialist services? | | | |
| Please add any additional comments (including related barriers, enablers and examples of best practice) | | | |
| **Staff training** | | | |
| 29. Practice / PCN / Trust staff access training to support them to work with people with severe mental illness, e.g. Making Every Contact Count training, training to support interventions related to smoking and alcohol. Annual physical health check staff training around engagement with patients living with a diagnosis of severe mental illness and supporting adoption of healthier lifestyle where possible | 9 | 9 | 1 |
| Please add any additional comments (including related barriers, enablers and examples of best practice) | | | |
| 30. Peer support workers and/or health and wellbeing coaches in the PCN, social prescribers and/or social workers are used to engage with those who are hard to reach and support those who are navigating pathways into community services and/or acute / specialist services for those identified as requiring support via their physical health checks | 11 | 8 | 0 |
| Please add any additional comments (including related barriers, enablers and examples of best practice) | | | |
