# Supplementary material for: Physical Health Checks and Follow‐Up Care in Deprived and Ethnically Diverse People With Severe Mental Illness: Co‐Designed Recommendations for Better Care
Source: Health Expect. 2024 Aug 28;27(5):e70005. doi: 10.1111/hex.70005 (PMC11350427; doi:10.1111/hex.70005)
Supplement: Supplementary file 2 — Supporting information. [file HEX-27-e70005-s002.docx]

**Appendix 2: Local Authority questionnaire and response counts**

| About you |
| --- |
| Your name |
| Your role in Tower Hamlets Local Authority |

| **Domains of best practice** | **Best practice implementation** | | |
| --- | --- | --- | --- |
|  | Fully (n) | Partially / Limited / No (n) | I do not know (n) |
| **The commissioning landscape across the Local Authority** | | | |
| 1. There is specific reference to residents with severe mental illness in the following Local Authority’s health improvement services and programmes: | | | |
| Stop smoking services | 1 | 0 | 2 |
| Substance misuse services | 0 | 1 | 2 |
| Physical activity programmes | 0 | 0 | 3 |
| Weight management programmes | 0 | 1 | 2 |
| Oral health improvement programmes | 0 | 0 | 3 |
| Please add any additional comments (including related barriers, enablers and examples of best practice) | | | |
| **System governance and leadership** | | | |
| 2. The Local Authority is part of the Integrated Care System governance structure for the physical health check programme for local residents with severe mental illness | 0 | 1 | 2 |
| Please add any additional comments (including related barriers, enablers and examples of best practice) | | | |
| 3. Improving the physical health of people with severe mental illness is included in local strategies and plans i.e. Health Inequalities Strategy, Public Mental Health Strategy, Health and Wellbeing Board Strategy | 0 | 3 | 0 |
| Please add any additional comments (including related barriers, enablers and examples of best practice) | | | |
| 4. There is a clear internal governance structure in place within the Local Authority with oversight of planned activity to improve outcomes for residents with severe mental illness | 1 | 2 | 0 |
| Please add any additional comments (including related barriers, enablers and examples of best practice) | | | |
| 5. There is a senior leader or champion within the Local Authority for improving the physical health of residents living with severe mental illness | 1 | 1 | 1 |
| Please add any additional comments (including related barriers, enablers and examples of best practice) | | | |
| 6. There is collaboration internally and with external partner organisations e.g. Adult Social Care, the Community, Voluntary and Social Enterprise sectors and the voices of lived experience | 1 | 2 | 0 |
| Please add any additional comments (including related barriers, enablers and examples of best practice) | | | |
| 7. There are explicit links with primary care, primary care networks and social prescribing in order to support interventions following the physical health checks for residents living with severe mental illness | 1 | 2 | 0 |
| Please add any additional comments (including related barriers, enablers and examples of best practice) | | | |
| **Commissioning and finances** | | | |
| 8. The commissioning arrangements are clear and transparently understood | 2 | 0 | 1 |
| Please add any additional comments (including related barriers, enablers and examples of best practice) | | | |
| 9. There is an identified lead officer or commissioner for improving the physical health of residents with severe mental illness in the Local Authority | 1 | 1 | 1 |
| Please add any additional comments (including related barriers, enablers and examples of best practice) | | | |
| 10. All relevant contracts include access and completion data for residents with severe mental illness. All specifications relevant to improving the physical health of residents with severe mental illness:   - have been reviewed in the last 2 years - have been co-produced - include data on referrals from primary care following a physical health check - include activity levels of interventions - include requirements about workforce planning and development - include information sharing/partnership working - include quality metrics - include outcomes | 1 | 1 | 1 |
| Please add any additional comments (including related barriers, enablers and examples of best practice) | | | |
| 11. Commissioners understand whether these services adequately cater to the needs of residents with severe mental illness | 1 | 2 | 0 |
| Please add any additional comments (including related barriers, enablers and examples of best practice) | | | |
| 12. Local Authority commissioners understand what the main barriers and facilitators are to accessing physical health checks and specifically the subsequent interventions | 1 | 2 | 0 |
| Please add any additional comments (including related barriers, enablers and examples of best practice) | | | |
| 13. Relevant services and programmes that would improve outcomes have been prioritised for review and improvement | 0 | 1 | 2 |
| Please add any additional comments (including related barriers, enablers and examples of best practice) | | | |
| **Data analysis and flows: understanding local need** | | | |
| 14. Health Impact Assessments, Health Equity Assessments and the Public Health England HEAT tool are utilised to better understand the needs of the local population, including those with protected characteristics, and enable effective targeting to ensure take up of physical health checks for those with severe mental illness | 2 | 0 | 1 |
| Please add any additional comments (including related barriers, enablers and examples of best practice) | | | |
| 15. Health Impact Assessments, Health Equity Assessments and the Public Health England HEAT tool are used to agree priorities for action | 0 | 2 | 1 |
| Please add any additional comments (including related barriers, enablers and examples of best practice) | | | |
| 16. The Local Authority and local commissioners have a clear understanding around the causes of death for people diagnosed with severe mental illness | 2 | 1 | 0 |
| Please add any additional comments (including related barriers, enablers and examples of best practice) | | | |
| 17. Local Authority consults with residents with severe mental illness to understand what helps and hinders them to improve their physical health | 1 | 2 | 0 |
| Please add any additional comments (including related barriers, enablers and examples of best practice) | | | |
| **Pathways** | | | |
| 18. The Local Authority works with key partners and people with lived experience to develop pathways into community-based services | 2 | 1 | 0 |
| Please add any additional comments (including related barriers, enablers and examples of best practice) | | | |
| 19. Pathways have been developed and commissioned across the Local Authority that meet the requirements of the community mental health framework in supporting all people with severe mental illness to access interventions to support their physical health, including but not limited to: | | | |
| Stop smoking services | 1 | 1 | 1 |
| Drug and alcohol services | 0 | 2 | 1 |
| Physical activity programmes | 0 | 2 | 1 |
| Weight management programmes | 0 | 2 | 1 |
| Oral health improvement programmes | 1 | 0 | 2 |
| Please add any additional comments (including related barriers, enablers and examples of best practice) | | | |
| **Interventions** | | | |
| 20. All specifications ensure staff access training to support them to deliver Making Every Contact Count approaches and Identification and Brief Advice for a range of health improvement areas e.g. substance misuse services deliver Smoking Cessation Identification and Brief Advice | 1 | 1 | 1 |
| Please add any additional comments (including related barriers, enablers and examples of best practice) | | | |
| 21. All specifications that help improve the physical health of residents with severe mental illness ensure that providers use culturally appropriate ways to promote access and deliver interventions e.g. access to interpreters, resources in multiple languages, etc. | 1 | 2 | 0 |
| Please add any additional comments (including related barriers, enablers and examples of best practice) | | | |
| 22. Providers use a variety of communication methods and different languages in order to reach all residents with severe mental illness who have been referred to local health improvement services | 1 | 2 | 0 |
| Please add any additional comments (including related barriers, enablers and examples of best practice) | | | |
| 23. The Local Authority has information for residents in a variety of formats and languages about how they can improve their physical health (including oral health) | 0 | 0 | 3 |
| Please add any additional comments (including related barriers, enablers and examples of best practice) | | | |
| 24. The Local Authority considers residents with severe mental illness in its digital poverty strategy and plans for digital inclusion | 0 | 2 | 1 |
| Please add any additional comments (including related barriers, enablers and examples of best practice) | | | |
| **Service user experience** | | | |
| 25. The experience of residents living with a severe mental illness is fully understood including:   - their experience of accessing services - their experience of the available health improvement services and programmes | 1 | 1 | 1 |
| Please add any additional comments (including related barriers, enablers and examples of best practice) | | | |
| 26. Service user feedback reflects the protected characteristics of the cohort and is regularly collected and analysed to understand what is most valued and what is frustrating to service users | 0 | 2 | 1 |
| Please add any additional comments (including related barriers, enablers and examples of best practice) | | | |
| 27. Residents with severe mental illness are involved in the co-production of:   - any Health Impact Assessments or Health Equity Assessments - resources and information relating to improving the physical health (including oral health) for residents with severe mental illness for residents, their family and staff - pathways - interventions and programmes | 0 | 1 | 2 |
| Please add any additional comments (including related barriers, enablers and examples of best practice) | | | |
